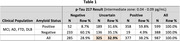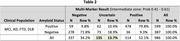# Multi‐Marker Approach to Reducing the Intermediate Range of a High Accuracy 2‐Cutoff Plasma p‐Tau 217 Test for Amyloid Detection

**DOI:** 10.1002/alz.095706

**Published:** 2025-01-09

**Authors:** David H Wilson, Meenakshi Khare, Michele Wolfe, Patrick Sheehy, Karen Copeland, Lyndal Hesterberg, Ann‐Jeanette Vasko, Wiesje M. van der Flier, Argonde C. van Harten, Inge M.W. Verberk, Charlotte Teunissen, Mike Miller

**Affiliations:** ^1^ Quanterix, billerica, MA USA; ^2^ Quanterix Corp, billerica, MA USA; ^3^ Quanterix Corp, Billerica, MA USA; ^4^ Boulder Statistics LLC, Boulder, CO USA; ^5^ HCS, Inc, Denver, CO USA; ^6^ Alzheimer Center Amsterdam, Neurology, Vrije Universiteit Amsterdam, Amsterdam UMC location VUmc, Amsterdam Netherlands; ^7^ Alzheimer Center Amsterdam, Amsterdam UMC, Amsterdam Netherlands; ^8^ Neurochemistry Laboratory, Department of Clinical Chemistry, Amsterdam Neuroscience, Program Neurodegeneration, Amsterdam UMC, Vrije Universiteit Amsterdam, Amsterdam, The Netherlands, Amsterdam Netherlands; ^9^ Neurochemistry Laboratory, Department of Clinical Chemistry, Amsterdam Neuroscience, Vrije Universiteit Amsterdam, Amsterdam UMC, Amsterdam Netherlands; ^10^ Quanterix Corporation, Billerica, MA USA

## Abstract

**Background:**

p‐Tau 217 is considered the most accurate single plasma biomarker for detecting amyloid pathology. Recent draft Alzheimer’s Association criteria for diagnosing Alzheimer’s recommends that p‐Tau 217 tests be designed with two cut‐offs in recognition of signal overlap between diseased and non‐diseased subjects. Two cutoffs maximizes the accuracy of the test, but leaves a diagnostic intermediate zone with uncertainty of amyloid status. It is desirable to minimize the indeterminant zone to reduce the number of patients receiving uncertain results. We investigated whether the inclusion of plasma amyloid b42/40 ratio, NfL, and GFAP into a multi‐marker logistic regression model including p‐Tau 217 would enable amyloid classification of uncertain results from p‐Tau 217 alone.

**Method:**

We optimized diagnostic thresholds for the Simoa LucentAD p‐Tau 217 plasma test to achieve ≥90% accuracy for MCI and AD patients across 2 independent cohorts (Bio‐Hermes, n = 242, Amsterdam Dementia Cohort, n = 737) representing divergent clinical settings, comparator methods, and geographic/ethnic/racial/mean age samplings. Across these diverse cohorts, the intermediate zone was 32.9%. Next we tested all samples for amyloid ratio, NfL, and GFAP using the Simoa N4PE kit. Multivariate logistic regression modeling was performed on all biomarker results to arrive at a probability scale. Samples with uncertain p‐Tau 217 results were re‐classified based on their probabilities being outside 90% lower and upper boundaries of the multi‐marker intermediate zone.

**Result:**

Tables 1 and 2 exhibit preliminary amyloid classification results for p‐Tau 217 alone and the multi‐marker model respectively. “Amyloid Status” was determined by amyloid PET (Bio‐Hermes) or CSF biomarkers (Amsterdam). The multi‐marker model enabled amyloid classifications for 189 previously uncertain results. 137 uncertain results were re‐classified as positive (accuracy 88%), and 52 uncertain results were re‐classified as negative (accuracy 87%), reducing the intermediate zone 2.4‐fold from 32.9% to 13.7%.

**Conclusion:**

Previous studies have found no benefit to combining additional AD‐relevant biomarkers with p‐Tau 217 to enhance accuracy. We show for the first time that additional biomarkers reduces the intermediate zone of a 2‐cutoff p‐Tau 217 test while preserving high accuracy. This finding can help provide amyloid status certainty for a greater number of symptomatic patients undergoing evaluation for Alzheimer’s.